# Geographic trends of scientific output and citation practices in psychiatry

**DOI:** 10.1186/s12888-014-0332-6

**Published:** 2014-12-06

**Authors:** Artemis Igoumenou, Klaus Ebmeier, Nia Roberts, Seena Fazel

**Affiliations:** Violence Prevention Research Unit, Wolfson Centre of Preventive Medicine, Barts and the London School of Medicine and Dentistry, Queen Mary, University of London, Garrod Building, Turner Street, London, E1 2AD England; Department of Psychiatry, Warneford Hospital, University of Oxford, Oxford, England; Bodlean Library, Oxford, England

**Keywords:** Scientific output, Citation practices, Psychiatry

## Abstract

**Background:**

Measures of research productivity are increasingly used to determine how research should be evaluated and funding decisions made. In psychiatry, citation patterns within and between countries are not known, and whether these differ by choice of citation metric.

**Method:**

In this study, we examined publication characteristics and citation practices in articles published in 50 Web of Science indexed psychiatric and relevant clinical neurosciences journals, between January 2004 and December 2009 comprising 51,072 records that produced 375,962 citations. We compared citation patterns, including self-citations, between countries using standard x^2^ tests.

**Results:**

We found that most publications came from the USA, with Germany being second and UK third in productivity. USA articles received most citations and the highest citation rate with an average 11.5 citations per article. The UK received the second highest absolute number of citations, but came fourth by citation rate (9.7 citations/article), after the Netherlands (11.4 citations/article) and Canada (9.8 citations/article).

Within the USA, Harvard University published most articles and these articles were the most cited, on average 20.0 citations per paper. In Europe, UK institutions published and were cited most often. The Institute of Psychiatry/Kings College London was the leading institution in terms of number of published records and overall citations, while Oxford University had the highest citation rate (18.5 citations/record).

There were no differences between the self-citation practices of American and European researchers.

Articles that examined some aspect of treatment in psychiatry were the most published. In terms of diagnosis, papers about schizophrenia-spectrum disorders were the most published and the most cited.

**Conclusions:**

We found large differences between and within countries in terms of their research productivity in psychiatry and clinical neuroscience. In addition, the ranking of countries and institutions differed widely by whether productivity was assessed by total research records published, overall citations these received, or citations per paper. The choice of measures of scientific output could be important in determining how research output translates into decisions about resource allocation.

## Background

Citation practices differ widely within and between countries, while citation rates are increasingly used by funding agencies, universities, and governments to determine grants, career pathways, and the scientific impact of individuals, research groups, and countries [1,2]. Within psychiatry, there has been research on local, national and international scientific productivity focusing on specific subspecialties and the most popular research areas within them [3-11], on individual institutions [12-14], journals [15,16], the contribution of different countries [8,17], practices [18] and early recognition of high quality researchers [19,20]. However, there has been little research examining trends in citation practice [21]. There are two reasons why examining citation trends is important. First, in determining how research should be evaluated, there are various models that need testing against each other. In some countries, it has been shown that there is a correlation between direct peer review of research groups and research bibliometrics [22] but this needs further examination within fields and between different bibliometric measures. Second, the practice of targeted self-citation is contentious, as it can be used to boost impact. Within some psychiatric subspecialties, it has been suggested that US authors disproportionately self-cite and cite colleagues from US institutions [23,24], a trend also reported in the wider scientific literature [25]. Furthermore, in general medicine [26] and in some specialties [27] it has been shown that US authors cite UK authors less often than UK authors cite US authors. We sought to examine whether these patterns are found in the field of psychiatric research.

## Methods

We examined publication characteristics and citation practices in articles published in 50 Web of Science indexed psychiatric and relevant clinical neurosciences journals, between January 2004 and December 2009. A time period of 5 years was considered to be long enough to provide a large number of articles. We collected our data in 2011; hence we allowed publications at least 2 years to receive citations. We selected all journals that have the root “psychiatr*” in the title (n = 34), as we wanted to exclude research published in other scientific fields such as neurology or neurosurgery. As not all psychiatric journals included reference to “psychiatry” in the title, we searched for all further journals that belonged to the categories of neurosciences, behavioural sciences, and psychiatry and extracted data of a further 16 general journals in these fields (Table 1). We chose to use Web of Science (within the Web of Knowledge), because of its potential to systematically provide data for informetric analysis, including publication and citation analysis, as well as the fact that it comprehensively includes databases, such as the Science Citation Index, the Social Sciences Citation Index, the Journal Citation Reports, and the Essential Sciences Indicators [4].

**Table 1:** **Journals included in the citation analyses according to total number of records published and total number of citations received (total global citation score, TGCS)**

**#**	**Journal**	**Records**	**TGCS**
1	Biological Psychiatry	6037	46779
2	European Psychiatry	4231	3764
3	Journal of Neurology, Neurosurgery and Psychiatry	3502	21992
4	American Journal of Psychiatry	2830	44413
5	Nervenarzt	2419	1697
6	Psychiatric Services	2206	8115
7	Journal of Clinical Psychiatry	2094	24443
8	Psychopharmacology	1971	27031
9	British Journal of Psychiatry	1912	16435
10	Addiction	1721	13910
11	Australian and New Zealand Journal of Psychiatry	1628	5031
12	Acta Psychiatrica Scandinavica	1615	8006
13	Progress in Neuro-Psychopharmacology & Biological Psychiatry	1147	8919
14	CNS Spectrums	1064	3851
15	Pharmacopsychiatry	1001	2904
16	International Journal of Geriatric Psychiatry	974	6549
17	Psychiatry Research	863	6829
18	Nervenheilkunde	827	572
19	Canadian Journal of Psychiatry	815	3516
20	Archives of General Psychiatry	806	32233
21	Psychiatry and Clinical Neurosciences	806	2646
22	Journal of Child Psychology and Psychiatry	704	11167
23	Molecular Psychiatry	680	19618
24	Nordic Journal of Psychiatry	666	1078
25	American Journal of Geriatric Psychiatry	665	6794
26	Psychosomatics	604	2704
27	Fortschritte der Neurologie Psychiatrie	598	785
28	Journal of Psychiatric Research	544	6023
29	Current Opinion in Psychiatry	470	3313
30	General Hospital Psychiatry	466	3086
31	CNS Drugs	453	5104
32	International Journal of Psychiatry in Clinical Practice	450	410
33	Neuropsychobiology	424	2834
34	Psychiatry-Research Neuroimaging	403	4772
35	Comprehensive Psychiatry	394	2647
36	European Archives of Psychiatry and Clinical Neuroscience	371	3420
37	Psychopathology	285	1358
38	Verhaltenstherapie	258	342
39	Neurocase	252	1190
40	Journal of Psychiatry & Neuroscience	240	2641
41	Archives of Psychiatric Nursing	228	507
42	Perspectives in Psychiatric Care	216	293
43	International Journal of Psychiatry in Medicine	209	741
44	World Journal of Biological Psychiatry	172	1095
45	World Psychiatry	170	552
46	Journal of Geriatric Psychiatry and Neurology	165	1324
47	Neuropsychiatrie	151	401
48	Neurology Psychiatry and Brain Research	148	85
49	International Journal of Methods in Psychiatric Research	132	1952
50	Primary Care and Community Psychiatry	85	82

At the time of our collection of data and analysis, the Web of Science did not provide the facility for direct analysis of citation practices in large datasets; hence we used HistCite, a complementary tool. We extracted all relevant articles with their references, citations and authors’ affiliations from Web of Science and imported the data into a software tool for informetric analysis of citation linkage, HistCite. HistCite provides citation linkage between scientific papers with tables and graphs that assist visualising the flow of publications and citations within a scientific field [4,28]. Using HistCite, we identified numbers of scientific publications for countries and institutions, characteristics of papers that attract more or fewer citations, and the flow of citations between different scientific publications.

With regard to institutions and countries that produce publications or receive citations, HistCite analyses data collected from the address field of the records in Web of Science. Institutions and countries, therefore, represent the affiliation of the corresponding author. As some of the institutions consist of a number of subdivisions, hence different authors’ affiliations, we explored whether institutions subsumed into greater local units were more productive and if their research was cited more often than others. We included all article types, such as original research, abstracts, reviews, and letters, taking into consideration that some article types attract more citations than others.

To examine citations practices amongst different countries and institutions, we used the Global Citation Score (GCS), which represents the total number of citations as given by Web of Science. From the GCS we also calculated the average number of citations per paper. Many measures have been used to quantify an individual researcher’s citation impact, some of which provide also information about the quality of the publications, such as the h-index and the g-index [29-31]. We chose the GCS over other metrics as we focused on the scientific productivity and impact at country and university level rather than at researcher level. A major advantage of the GCS scores is that they are readily available from the Web of Science, as is the h-index. Citations are, of course, only one measure of scientific impact and do not distinguish between the quality of the publication, the impact of the journal citing and whether the citation is positive or negative. Citations have some of the limitations of the other measures of academic output. As with other measures, citations can be magnified or deflated according to researchers productivity concerning articles included in WoS database [31], their scientific collaborations and can be influenced by individuals in the same institution or group of institutions citing each other. Citations can potentially be exposed to errors due to articles being cited in different ways, variations in author affiliations or multiple author affiliations, and variations in institutional naming or indeed omission of country/institute of origin [32,33].

As HistCite does not automatically provide information about the details of citation practices, such as the distribution of citations between countries and institutions, and the number of self-citations, we extracted this information manually in a stratified sample of 1000 records. In order to gain representation of our initial sample of 51,072 articles, we collected the first 500 articles from the USA (as presented on HistCite in chronological order), and equally the first 500 articles from Europe (200 records from UK, 200 from Germany and 100 from other European countries). We sampled the earliest articles included in our initial search, as they had a better chance to generate citations since being published. For the purposes of this sub-study, we used the Local Citation Score (LCS), which is the number of citations that a paper received within the field of psychiatry. Since research collaboration between countries is an increasing phenomenon, we also investigated the proportion of the publications in our sample that resulted from such collaboration. In a sensitivity analysis we combined Canada with the US (500 articles) to make North America and we investigated citation practices in North America and Europe.

We furthermore attempted to identify subject trends by examining the most common key words in the titles of publications. HistCite automatically generates results on the most common key words as they were identified by the article authors.

We investigated the following questions: Which country publishes more and which country’s research attracts most citations? What are the world’s top ten institutes in psychiatry in terms of publication productivity? What are the subjects in psychiatry that generate more publications? Who cites whom in psychiatry (self citations, within and between countries citation trends)? We used standard χ^2^ tests to test for differences between proportions.

## Results

The 50 included psychiatric journals published a total of 51,072 articles between January 2004 and January 2009, most of which (n = 46,984, 92%) were published in English. These articles were written by 82,092 authors.

We found that most publications came from the USA, with Germany being second and UK third in publication productivity (Table 2). It was also evident that when counting the total number of citations (Total Global Citation Scores or TGCSs), USA articles received most citations and the highest citation rate with an average 11.5 citations per article. The UK received the second highest absolute number of citations, but came fourth by citation rate (9.7 citations/article), after the Netherlands (11.4 citations/article) and Canada (9.8 citations/article).

**Table 2:** **Rankings in psychiatry/neurosciences ordered by total number of citations received (total global citation score, TGCS) (ranking in brackets)**

**#**	**Country**	**TGCS**	**Records**	**Citations/paper**
1	USA	202781 (1)	17610 (1)	11.5 (1)
2	UK	62486 (2)	6468 (3)	9.7 (4)
3	Germany	35526 (3)	7095 (2)	5.0 (9)
4	Canada	24041 (4)	2451 (5)	9.8 (3)
5	Netherlands	19206 (5)	1686 (7)	11.4 (2)
6	Australia	17734 (6)	2192 (6)	8.1 (6)
7	Italy	14476 (7)	1568 (9)	9.2 (5)
8	Japan	10341 (8)	1616 (8)	6.4 (8)
9	Switzerland	9643 (9)	1377 (10)	7.0 (7)
10	Unknown	3733 (10)	4855 (4)	0.8 (10)

Both globally and within the USA, Harvard University published most articles and these articles were the most cited, on average 20.0 citations per paper (see Table 3 for the top ten world institutions and Table 4 for the top ten USA institutions in terms of the citations received).

**Table 3:** **Rankings of top 10 research institutions ordered by total number of records produced (ranking in brackets)**

**#**	**Institution**	**TGCS**	**Records**	**Citations/paper**
1	Harvard Univ	38910 (1)	1948 (1)	20.0 (1)
2	Inst Psychiatry	16730 (2)	1346 (2)	12.4 (8)
3	Univ Pittsburg	15420 (3)	970 (3)	15.9 (6)
4	Yale Univ	14633 (4)	894 (4)	16.4 (4)
5	Columbia Univ	14381 (6)	882 (5)	16.3 (5)
6	NIMH	14451 (5)	810 (6)	17.8 (2)
7	Toronto Univ	8408 (9)	743 (7)	11.3 (9)
8	Univ Calif Los Angeles	12634 (7)	741 (8)	17.1 (3)
9	Univ Calif San Diego	9942 (8)	716 (9)	13.9 (7)
10	Univ Munich	6010 (10)	651 (10)	9.2 (10)

**Table 4:** **Rankings of USA research institutions ordered by total number of citations received (total global citation score, TGCS) (ranking in brackets)**

**#**	**Institution**	**TGCS**	**Records**	**Citations/paper**
1	Harvard Univ	38910 (1)	1948 (1)	20.0 (1)
2	Univ Pittsburg	15420 (2)	970 (2)	15.9 (9)
3	Yale Univ	14633 (3)	894 (3)	16.4 (5)
4	NIMH	14451 (4)	810 (5)	17.8 (2)
5	Columbia Univ	14381 (5)	882 (4)	16.3 (6)
6	Univ Calif Los Angeles	11802 (6)	741 (6)	15.9 (8)
7	Univ Texas	11322 (7)	650 (8)	17.4 (3)
8	Univ Calif San Diego	9942 (8)	716 (7)	13.9 (10)
9	Univ Penn	9029 (9)	565 (9)	16.0 (7)
10	Duke Univ	8830 (10)	517 (10)	17.1 (4)

In Europe, UK institutions published and were cited most often. We found that the Institute of Psychiatry/Kings College London was the leading institution in terms of number of published records and overall citations, while Oxford University had the highest citation rate (18.5 citations/record; Table 5 for the ranking of the top ten European research institutions and Table 6 for the ranking of the top ten UK research institutions).

**Table 5:** **Rankings of European research institutions ordered by total number of citations (total global citation score, TGCS) (ranking in brackets)**

**#**	**European Centre**	**TGCS**	**Records**	**Citations/paper**
1	Kings Coll/IoP	16730 (1)	1346 (1)	12.4 (7)
2	Univ Oxford	6497 (2)	352 (4)	18.5 (1)
3	Univ Cambridge	6270 (3)	340 (6)	18.4 (2)
4	Univ Munich	6010 (4)	651 (2)	9.2 (9)
5	UCL	4660 (5)	407 (3)	11.5 (8)
6	Univ Manchester	3912 (6)	301 (7)	13.0 (6)
7	Univ Utrecht	3209 (7)	230 (8)	14.0 (5)
8	Karolinska Inst	3151 (8)	342 (5)	9.2 (10)
9	Maastricht Univ	2799 (9)	193 (9)	14.5 (4)
10	Vrije Univ Amsterdam	2750 (10)	170 (10)	16.2 (3)

**Table 6:** **Rankings of UK research institutions by citations received ordered by total number of citations received (total global citation score, TGCS) (ranking in brackets)**

**#**	**UK Institution**	**TGCS**	**Records**	**Citations/paper**
1	Kings College London/Institute of Psychiatry	16730 (1)	1346 (1)	12.4 (7)
2	Univ Oxford	6497 (2)	352 (3)	18.5 (2)
3	Univ Cambridge	6270 (3)	340 (4)	18.4 (3)
4	University College London	4660 (4)	407 (2)	11.5 (8)
5	Univ Manchester	3912 (5)	301 (5)	13.0 (5)
6	Univ Bristol	2592 (6)	181 (6)	14.3 (4)
7	Univ Edinburgh	2167 (7)	171 (7)	12.7 (6)
8	Univ Southampton	2068 (8)	111 (10)	18.6 (1)
9	Univ Newcastle Upon Tyne	1446 (9)	135 (9)	10.7 (9)
10	Univ London Imperial	1355 (10)	144 (8)	9.4 (10)

We explored whether institutions subsumed into greater local units were more productive and if their research was cited more often than others (see Table 7 for European institutions). The rankings of the institutions were broadly confirmed when the various subdivisions were combined.

**Table 7:** **Rankings of European research institutions by subdivision ordered by total number of citations received (total global citation score, TGCS) (ranking in brackets)**

**#**	**Institutions**	**TGCS**	**Records**	**Citations/paper**
1	Kings College London/Institute of Psychiatry	9130 (1)	718 (1)	12.7 (7)
2	Univ Cambridge, Dept Psychiat/Addenbrokes Hospital	4210 (2)	196 (3)	21.5 (3)
3	Univ Oxford, Dept Psychiat/Warneford Hospital	4026 (3)	173 (4)	23.3 (1)
4	Univ Munich, Dept Psychiat	3302 (4)	292 (2)	11.3 (8)
5	Inst Psychiat, Div Psychol Med	2212 (5)	124 (5)	17.8 (5)
6	Univ Utrecht, Med Ctr	1831 (6)	101 (7)	18.1 (4)
7	Univ Cambridge, Dept Expt Psychol	1516 (7)	68 (10)	22.3 (2)
8	Maastricht Univ, Dept Psychiat & Neuropsychol	1371 (8)	77 (9)	17.8 (6)
9	Univ Amsterdam, Acad Med Ctr	1097 (9)	100 (8)	11.0 (9)
10	Leiden Univ, Med Ctr	1056 (10)	103 (6)	10.3 (10)

HistCite does not provide direct information about flow of citations among different countries, nor has information on self-citation rates. Therefore, we collected this information manually from a stratified sample of 1,000 articles.

As shown on Table 8, the selected 1,000 records were cited on 3504 occasions. Seventy-one per cent (n = 2,488) of citations came from the USA and 29% (n = 1,016) from Europe. We found that Americans cite Americans more often (50%, n = 1,252) and Europeans cite more European papers (59%, n = 596) (χ^2^ = 336, d.f. = 1, p < 0.001). Both Americans and Europeans receive citations from the rest of the world less often than from their own continent (n = 443, 18% and n = 193, 18% respectively; χ^2^ = 334, d.f. = 2, p < 0.001; Table 8).

**Table 8:** **Citations received by country in a subgroup of 1000 articles originated from USA (n = 500) and Europe (n = 500)**

	**USA**	**Europe**	**Rest of the World**	**Anonymous**	**Total citations (n = 3504)**
**USA**	1240 (0.50)	712 (0.29)	443 (0.18)	88 (0.03)	2488 (0.71)
**Europe**	199 (0.20)	596 (0.59)	193 (0.18)	28 (0.03)	1016 (0.29)

Note: Columns are the countries from which citation donors are based; rows are the countries which receive citations. The 1000 articles considered received 3504 citations.

When counting separately for UK publications (n = 693), we found that British publications more often cited other Europeans (30%), whilst cited American (22%) and British (23%) records equally often. UK articles constituted only 6% of the American and 17% of other European citations.

We also explored numbers of self-citations. From the total of 3,504 citations that our 1,000 records received, 710 (20%) were self-citations. We found no difference between the self-citation practices of the Americans (20% of their citations were self-citations) and the Europeans (21% were self-citations) (χ^2^ = 0.001, d.f. = 1, p = 0.974). We also found that UK researchers were less likely to self-cite compared to all other countries, only 15% of their citations were self-citations (χ^2^ = 8.7, d.f. = 1, p = 0.032).

In addition, we investigated how many records of the 3,504 publications were international research collaborations and found that 22% (n = 755) were collaborations between two or more countries.

In a sensitivity analysis, we combined Canada and the US to make North America and we found no material differences in citing papers from one’s own continent (χ^2^ = 314, d.f. = 1, p < 0.001) and in self-citation practices (χ^2^ = 0.01, d.f. = 1, p = 0.919) compared with the above analysis (Table 9).

**Table 9:** **Sensitivity analysis for citations received by country in a subgroup of 1000 articles originated from North America (n = 500) and Europe (n = 500)**

	**North America**	**Europe**	**Rest of the world**	**Anonymous**	**Total citations (n = 3393)**
North America	1294 (0.54)	675 (0.28)	324 (0.14)	84 (0.04)	2377 (0.70)
Europe	248 (0.24)	596 (0.59)	144 (0.14)	28 (0.03)	1016 (0.30)

Note: Columns are the countries from which citation donors are based; rows are the countries which receive citations. The 1000 articles considered received 3393 citations.

We found that North Americans cite North Americans more often (54%, n = 1,294) and Europeans cite more European papers (59%, n = 596) (x^2^ = 314, d.f. = 1, p < 0.001). Both Americans and Europeans receive citations from the rest of the world less often than from their own continent (x^2^ = 314, d.f. = 2, p < 0.001).

We also explored numbers of self-citations. From the total of 3,393 citations that our 1,000 records received, 691 (20%) were self-citations. We found no difference between the self-citation practices of the Americans (20% of their citations were self-citations) and the Europeans (21% were self-citations) (x^2^ = 0.01, d.f. = 1, p = 0.919).

In addition, we investigated how many records of the 3,393 publications were international research collaborations and found that 22% (n = 746) were collaborations between two or more countries.

The above results are similar to those between US and Europe (Table 8). It seems that considering North America (US and Canada) rather than US alone does not materially alter our conclusions.

With regards to subject trends we found that articles focusing on treatment (including key words such as therapy, care, and management) were the most published (n = 8,737). In terms of diagnosis, schizophrenia-related topics had the most publications (n = 5,855 records) and were the most cited (TGCS = 51,842). Articles on depressi* (n = 5,120), together with articles on bipolar (n = 2,185) and cogniti* (n = 2,098) were also common keywords for articles.

## Discussion

Journal impact factors and citation practices are increasingly used in the evaluation of research productivity and quality in medicine and other fields. In psychiatry, there have been bibliometric studies on scientific productivity focusing on specific subspecialties and the most popular research areas within them [5-11], on individual institutions [14], the contribution of different countries [8,17], practices [18] and early recognition of high quality researchers [19]. However, to our knowledge, there has not been an overall examination of citation practices in psychiatry and clinical neurosciences.

This report presents the findings of research productivity across countries and institutions by tracking citations numbers and rates in psychiatry and related neuroscience. We examined 51,072 records that produced 375,962 citations during 2004–2009. There are two main findings. First, across countries, the findings underscore the dominance of US research in these fields. In relation to citation counts, US research produced more citations than the next nine most productive countries combined. This dominance was less obvious in relation to citation rates (citations per publication), although there appeared to be large differences between countries. One notable difference was that between Germany and the Netherlands, where citation rates differ two-fold (citations per publication 5.0 and 11.4 respectively). Second, within countries, there was a disparity in the top 10 most productive institutions depending on whether the number of publications, their citations, or their citation rate was counted. In other words, institutional research impact appears to differ markedly, depending on what output was used.

Our findings may have implications for the development of research environments and their impact on productivity. The extent to which language, research governance, and national funding determine these differences needs further examination. The UK, in particular, has spent considerable resources developing a model of peer review that determines research funding for many years. We found differences in institutional impact by total numbers of records, citations, and citations per article. When applied to funding decisions this could lead to large differences in core funding for these research institutions. A final observation is the relative under-representation on non-European and non-American research groups in this field, a problem apparent in wider science [3]. This observation is consistent with other research on global representation in high impact psychiatric research, showing that psychiatric research published in high impact psychiatric journals was generated mainly in regions that represented only a small part of world’s population [4].

In a sub-study, we tracked citations between countries and self-citation rates. We found that citation rates appeared to be broadly distributed according to article productivity. There are two possible implications of this. First, there is a certain and understandable preference in psychiatry and clinical neurosciences for research from an author’s own country that can be found equally on both sides of the Atlantic. This may be explained by the author’s deciding on citing material from their national journals [17], which would tend to be populated by local researchers, rather than any other reason. Second, researchers in the field need to consider whether this potentially limits the interpretation and generalizability of their work. Interestingly, we found no strong differences in self-citation rates between Europe and US.

We performed a sensitivity analysis to investigate whether the results of our sub-study applied to North America (Canada and US). We looked at 1000 articles, 500 from the North America (US and Canada) and 500 from Europe. We found no material differences in citation practices (self-citation, preferences in research from an author’s own continent) compared with the results of our sub-study.

In the same sub-study we found that 22% (n = 755) of the citations were collaborations between two or more countries. Future research could explore whether researchers that collaborate internationally tend to cite other international collaborations.

One strength of the current report is that we have used a large sample of articles from a broad range of relevant journals - 51,072 articles published in 50 indexed psychiatric and clinical neurosciences journals during a five-year period (2004–2009). However, there are a number of important limitations. First we collected our data from Web of Science, hence we will be missing the articles published in journals excluded from it. We carefully avoided to compare the Web of Science with other databases used for citation analysis, such as the Scopus or the Google Scholar, as they are all using different sources to generate their data, hence are not directly comparable. Similarly, research on which database is preferable remains controversial [32,34,35]. We limited our study to the top 50 journals in psychiatry, which would miss the leading journals in important related fields such as nursing, social work and clinical psychology. Alternative approaches to selecting journals can be considered in future research. Another limitation is that we relied, for practical reasons, on the affiliation of the corresponding author to determine the location of the author and subsequent citation rates of their institution. We might therefore have underestimated the productivity of some collaborating institutions and countries. We did not explore the citation practices between authors and their scientific network, and were unable to examine how this would affect the flow of citations among researchers [36]. In our methods, we looked at all countries but decided to limit our results to the top 10 in research productivity. This resulted in countries represented in this paper to be predominantly Western. This is likely to change over time as some countries’ research infrastructure (such as China) develops. A large number (n = 4855) of articles could not be categorised by country. This is an important limitation of using a web-based data base to collect our data. It is not certain whether the articles that we could not categorise would be disproportionately from one country, and therefore the effect on the results is not known. In our sub-study we looked at 1000 articles, 500 from the US and 500 from Europe. This was an arbitrary decision based on where the main concentrations of research-active universities were. Thus this sub-study was limited in not being to assess important research countries such as Canada or Australia. Future work should include other countries such as Canada, Australia, and China, as their impact is increasingly recognised. We also used the oldest articles for the sub-study. It is possible that the trends are changing or will change in the future, especially with regards to the recent appearance of eastern countries in the citation map. Finally, we included all types of publications and this can be a limitation of our study as some types of articles attract more citations than others.

## Conclusions

In summary, in this large study of scientific output and citation trends in psychiatry and clinical neuroscience, we have found large differences between and within countries in terms of their research productivity. The choice of output will be important in determining how this translates into decisions about resource allocation for research.
